# Use of Multiflora Bee Pollen as a Flor Velum Yeast Growth Activator in Biological Aging Wines

**DOI:** 10.3390/molecules24091763

**Published:** 2019-05-07

**Authors:** Pau Sancho-Galán, Antonio Amores-Arrocha, Ana Jiménez-Cantizano, Víctor Palacios

**Affiliations:** Department of Chemical Engineering and Food Technology, Faculty of Sciences, University of Cadiz, Agrifood Campus of International Excellence (ceiA3), IVAGRO, P.O. Box 40, 11510 Puerto Real, Cadiz, Spain; pau.sancho@uca.es (P.S.-G.); ana.jimenezcantizano@uca.es (A.J.-C.); victor.palacios@uca.es (V.P.)

**Keywords:** Bee pollen, biological aging, activator, sherry wine

## Abstract

Flor velum yeast growth activators during biological aging are currently unknown. In this sense, this research focuses on the use of bee pollen as a flor velum activator. Bee pollen influence on viable yeast development, surface hydrophobicity, and yeast assimilable nitrogen has already been studied. Additionally, bee pollen effects on the main compounds related to flor yeast metabolism and wine sensory characteristics have been evaluated. “Fino” (Sherry) wine was supplemented with bee pollen using six different doses ranging from 0.1 to 20 g/L. Its addition in a dose equal or greater than 0.25 g/L can be an effective flor velum activator, increasing yeast populations and its buoyancy due to its content of yeast assimilable nitrogen and fatty acids. Except for the 20 g/L dose, pollen did not induce any significant effect on flor velum metabolism, physicochemical parameters, organic acids, major volatile compounds, or glycerol. Sensory analysis showed that low bee pollen doses increase wine’s biological aging attributes, obtaining the highest score from the tasting panel. Multiflora bee pollen could be a natural oenological tool to enhance flor velum development and wine sensory qualities. This study confirms association between the bee pollen dose applied and the flor velum growth rate. The addition of bee pollen could help winemakers to accelerate or reimplant flor velum in biologically aged wines.

## 1. Introduction

Biological aging is a microbiological process where a natural biofilm of a *Saccharomyces* yeast strain called flor velum develops on wine surface, resulting in Fino or Manzanilla wines [1,2]. Also, in some wine producing areas of the world such as Sardinia (Italy), Jura (France), Montilla-Moriles (Spain), and Tokay (Hungry), special wines are produced by biological aging processes [3]. Sherry flor velum are a film forming culture 95% composed of different *Saccharomyces cerevisiae* strains, growing under hard conditions such as low oxygen concentrations, high ethanol concentrations (from 15% to 16% *v*/*v*), low pH levels, and low fermentable sugar concentrations [4,5]. The flor velum develops an oxidative metabolism, where ethanol consumption as a primary carbon source has been widely studied, as well as glycerol, acetic, and lactic acid [6]. In this way, acetaldehyde, 2,3-butanediol, fusel alcohols, diacetyl, and acetoin among others are formed as reaction products [2,4,7,8]. For cell development and protein synthesis [9,10,11], flor velum yeasts use nutrients and growth factors such as nitrogen compounds, fatty acids, vitamins, etc., taken mainly from wine [12,13,14,15]. Scarce studies have been conducted on the relationship of the wine nitrogen content and composition, and the development of flor velum yeasts. Some authors highlighted the importance of amino acid consumption and production for the cellular redox potential balance [16]. In addition, Berlanga et al. (2006) [10] studied different processes such as the amino acid conversion into other more reduced, major alcohols, acids, and other compounds during biological aging. Flor velum yeasts can synthesize or release new amino acids during the biological aging process [16], and L-proline is the main source of nitrogen for flor velum yeasts during biological aging [6]. Nowadays, there is only one technique to activate the flor velum growth, and is based on the aeration and introduction of new wine in oak casks in order to introduce oxygen and nutrients [17]. Furthermore, in the current oenological market, there is a wide variety of amino acids and ammonium based products designed to supply grape musts with nutritional deficiencies [11,18], but there are not such products for the biological aging process.

In this regard, it should be noted that multiflora bee pollen could be a natural product that could fill the biological aging activation gap. Bee pollen is a natural product that comes from beehives and is rich in carbohydrates, lipids, amino acids, proteins, minerals, fatty acids, polyphenols, sterols, and phospholipids among other compounds of interest [19,20,21]. Previous research works showed how the application of high multiflora bee pollen doses produced a significant yeast assimilable nitrogen (YAN) increase in mead and white grape musts [18,22], decreasing the yeast lag phase during the alcoholic fermentation. This may be due to the fact that YAN is essential for the optimal growth and development of yeasts and it includes amoniacal nitrogen, amino acids, small peptides, and nitrogen that can be easily assimilated by yeasts [23].

For these reasons, the aim of this study is to evaluate the use of multiflora bee pollen as an activator of the biological aging process under flor velum and its influence on viable yeast population development, surface hydrophobicity or ability to float, and wine YAN content. In addition, the main parameters and compounds related to flor velum yeast metabolism and the typical sensory attributes of wines supplemented with pollen have been assessed.

## 2. Results

### 2.1. Influence of Bee Pollen on Flor Velum Yeast Development and Yeast Assimilable Nitrogen (YAN)

**Figure 1:**
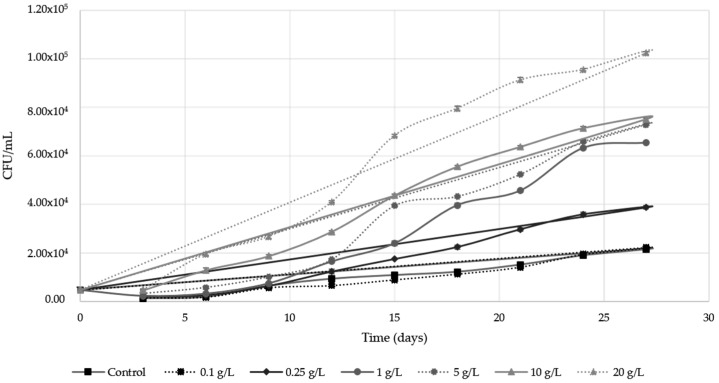
Evolution of the viable biomass of flor velum yeasts during the biological aging process of Palomino Fino wine with bee pollen doses. The results are the mean ± SD of three repetitions.

Figure 1 shows flor velum viable yeast evolution during the biological aging in multiflora bee pollen supplemented wines. Except for 0.1 g/L dose, bee pollen addition increased significantly total viable yeast cells in respect to the control, and a linear correlation between the bee pollen dose applied and the maximum viable yeast cells was observed (R^2^ = 0.94). As it can be seen, from the 5 g/L bee pollen dose on, flor velum yeast latency time was significantly reduced (3 days) compared to the control (5 days) (Figure 1). Both the control and 0.1 g/L dose samples presented a similar behavior, with a constant linear growth (R^2^ = 0.984 and R^2^ = 0.964 for the control and 0.1 g/L dose, respectively). At the 0.25 g/L dose the evolution was also linear, although a greater slope was observed in respect to the control and 0.1 g/L dose. Nevertheless, from the 5 g/L bee pollen dose on, an exponential yeast growth was observed until the 15th–18th days. After those days, the yeast growing rate slowed gradually until the end of the experiment. From the 18th day onwards, all doses except 0.1 g/L showed significantly higher population values in respect to the control (ANOVA *p* < 0.05), hence, the concentration of viable flor velum yeasts was 100–200% higher than the control for doses ranging between 0.25–20 g/L respectively. In this way, multiflora bee pollen was shown as an effective activator for development of flor velum yeasts from concentrations equal or greater than 0.25 g/L.

**Figure 2:**
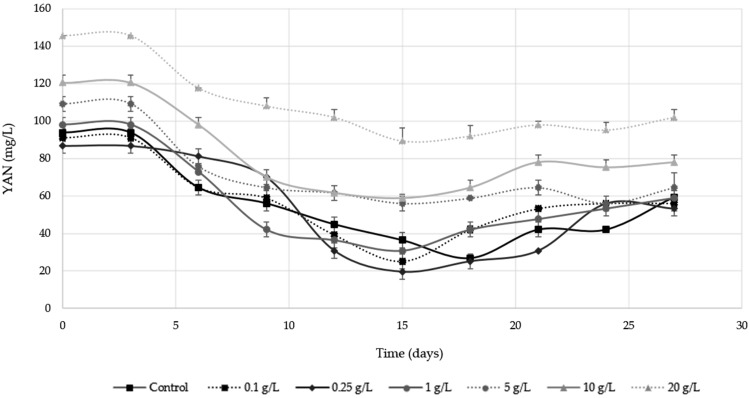
Evolution of the yeast assimilable nitrogen (YAN) by flor velum yeasts during the biological aging process of Palomino Fino wine with bee pollen doses. The results are the mean ± SD of three repetitions.

**Figure 3:**
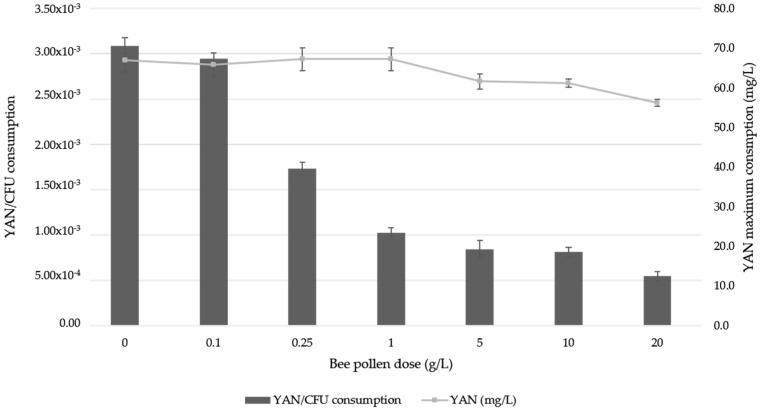
Maximum consumption of yeast assimilable nitrogen (YAN) per colony forming unit (CFU) during the exponential growth phase of Palomino Fino wine with different bee pollen doses and control.

Figure 2 shows the evolution of YAN content in wines during biological aging. As it can be seen, the effect of bee pollen on the initial wine YAN concentration was remarkable, increasing linearly with the bee pollen dose applied (R^2^ = 0.98). Once the biological aging process and yeast growth phase started, a significant YAN decrease was observed in all wines including the control, reaching the minimum YAN concentration at the 15th day (Figure 2). YAN content fall was more remarkable for the control and low-intermediate doses (0.1–1 g/L) with an average net reduction of 70%, than for medium or high doses (5–20 g/L), where the maximum reduction of YAN was less than 50%. This effect leads to the YAN consumption per colony forming unit (CFU) at the 15th–18th days decreasing significantly with the addition of bee pollen compared to control, with a maximum net reduction up to 60% for the 20 g/L dose (Figure 3). Once YAN values reached a minimum for each dose, its content started to increase, coinciding with the flor velum yeast stationary phase in most cases (Figure 1). At the end of the biological aging trials, all wines had a 37% lower YAN content than at the beginning of the experiment, and only the 10 and 20 g/L bee pollen doses showed significant differences (ANOVA *p* < 0.05) in respect to the control and the rest of the doses. 

### 2.2. Influence of Bee Pollen on Flor Velum Hydrophobicity during Biological Aging

**Figure 4:**
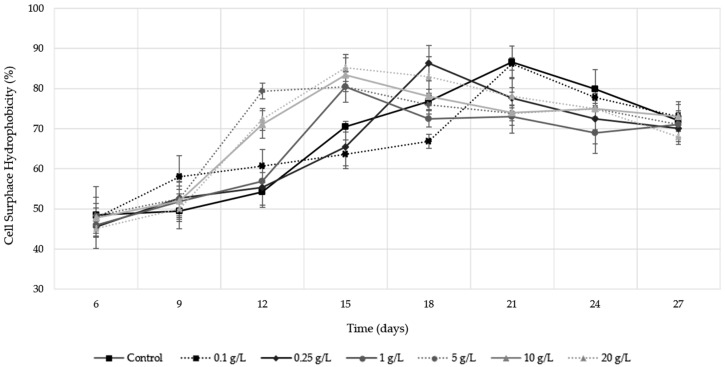
Cell surface hydrophobicity levels during the biological aging of Palomino Fino wine with bee pollen doses. The results are the mean ± SD of three repetitions.

Figure 4 shows flor velum yeast surface hydrophobicity evolution during biological aging. The evolution of surface hydrophobicity was related to the flor velum population development, and for this reason, cell surface hydrophobicity values could not be obtained until the 6th day. As expected, the hydrophobicity increase was exponential during the first 15 days for the pollen doses between 1 and 20 g/L, corresponding with the yeast exponential growth phase (Figure 1). Hydrophobicity levels reached in those doses were significantly higher (20–30%) compared to the control (ANOVA *p* < 0.05). Control and low bee pollen doses showed a linear growth trend, and the maximum values reached were not significantly different from wines with intermediate or high bee pollen doses (ANOVA *p* < 0.05). In addition, in the last days of biological aging, a decrease in the surface hydrophobicity values was observed, coinciding with the yeast stationary growth phase (Figure 1). Therefore, bee pollen addition (≥1 g/L) produces a significant increase in flor velum surface hydrophobicity during the exponential growth phase, giving stability and buoyancy to the velum.

### 2.3. Influence of Bee Pollen on Flor Velum Yeast Metabolism

Table 1 shows the influence of bee pollen addition on some compounds and parameters involved in flor velum metabolism. Regarding pH and total acidity no significant differences were found between the different bee pollen doses applied. Only a slight increase in pH for the 20 g/L bee pollen dose was observed. By comparing the ethanol values observed in Table 1 with those obtained by the potassium sorbate control, where no biological aging existed (13.38% ± 0.18), it can be determined that the flor velum yeast consumption only represents approximately 3.7% of the total decrease. Acetic acid represents another metabolite that flor velum consumes. As it can be seen in Table 1, volatile acidity presents an oscillatory behavior. Low and intermediate doses of bee pollen showed a significant decrease in volatile acidity values compared to the initial sample while for control and high doses (10–20 g/L) there was essentially no variation. Regarding organic acids composition, citric acid present in final wines ranged between 0.015 and 0.019 g/L (control and 20 g/L bee pollen, respectively). These values present a positive correlation between the dose of bee pollen applied and the concentration of this metabolite, with significant differences from the 5 g/L dose on (ANOVA, *p* < 0.05). In respect to tartaric acid values, these oscillated between 1.67 g/L (20 g/L bee pollen) and 2.02 g/L (control), with no significant differences between samples and control (Table 1). On one hand, malic acid did not show significant differences between the bee pollen samples and control. On the other hand, lactic acid content decreased significantly for all samples compared to the initial sample (ANOVA, *p* < 0.05). Finally, succinic acid was found in wines between 0.285 and 0.194 mg/L (control and the 20 g/L bee pollen, respectively). Although a 33% lower content than in the initial sample was observed, no significant differences were found between the different doses of bee pollen and the control. This last acid shows, unlike the previous ones, a negative correlation since it occurs at a lower concentration with a greater addition of bee pollen to wine.

Regarding the variation in the major volatile compounds content, a significant increase in acetaldehyde content was observed for all samples in respect to the initial simple. As it can be seen, acetaldehyde levels were significantly higher (between 15–53%) in wines supplemented with bee pollen in respect to the control, except for the highest dose (20 g/L) where the concentration reached was lower (Table 1). In relation to ethyl acetate concentration in wines, all the samples presented values between 52.71 mg/L (control) and 77.29 mg/L (20 g/L bee pollen); a significant relationship with acetic acid content was found [24]. In relation to methanol content, an increase of its concentration in respect to the control was observed showing significant differences from the 1 g/L dose on.

In respect to major alcohols (isobutanol, 1-propanol, and isoamyl alcohol) the same behavior was observed for the three alcohols studied up to the 5 g/L bee pollen dose sample. Low and intermediate bee pollen doses increased the major alcohol concentration in wines up to 52% compared to the initial sample. However, for the 20 g/L bee pollen dose no changes were observed in respect to the initial sample. Finally, glycerol content decreased significantly for all the samples in respect to the initial one. In addition, only the 20 g/L dose showed a significant decrease in respect to the rest of the wines after the biological aging.

### 2.4. Descriptive Sensory Analysis

**Figure 5:**
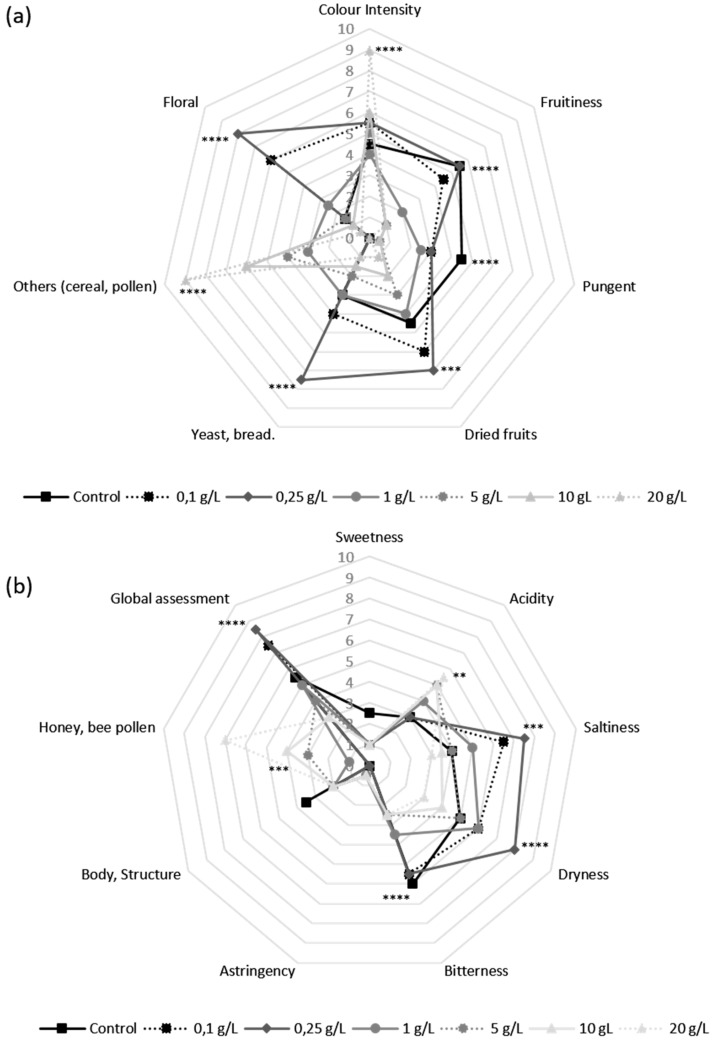
Bee pollen effect on the visual and olfactory (**a**) and taste (**b**) evaluation of Palomino Fino wine after biological aging. Stars indicate level of significance for two-way ANOVA according to Bonferroni’s multiple range test (BSD) (** *p* < 0.01, *** *p* < 0.001 and **** *p* < 0.0001).

To obtain a description of some of the wine organoleptic parameters, a sensory analysis of wines supplemented with pollen and the control was conducted. Spider web diagrams show the mean values for the attributes analyzed with the level of significance of control wine and samples with pollen. Figure 5 shows the results of visual and olfactory (a) and taste phase (b) respectively, as well as the global assessment of all wines tasted (b).

Significant differences were found for almost all the attributes studied. Color intensity showed significant differences with all the samples and the control for the 20 g/L bee pollen dose (Figure 5a). Regarding floral aroma, only 0.1 and 0.25 g/L doses showed significant differences from the rest of the samples and the control. In respect to the pollen/cereal attributes, a positive correlation was found between the dose of pollen applied and the intensity of these attributes, and only from 1 g/L doses on, significant differences were found in respect to the control and low doses (0.1–0.25 g/L) (Figure 5a). Biological aging typical aromas such as yeast or dried fruit showed higher values for low bee pollen doses (0.1 and 0.25 g/L), and only the 0.25 g/L dose showed significant differences in respect to the control and the rest of the doses. In turn, attributes related to biologically aged wines, such as pungency, decreased with pollen contribution, showing its maximum intensity in control wine (Figure 5a). The opposite happens with the pollen/honey flavor perception (Figure 5b), which are significantly increased from 5 g/L doses on and affect wine sensory qualities. No significant differences were found for the sweetness, astringency, and body/structure attributes (Figure 5b). According to the global assessment results, low doses of bee pollen (0.1–0.25 g/L) have been found to be optimal for the sensory profile improvement, showing significant differences in respect to the control and the rest of the bee pollen doses (ANOVA, *p* < 0.0001).

## 3. Discussion

According to the results, pollen addition in doses equal or greater than 0.25 g/L produces a significant increase in flor velum yeast populations during biological aging (Figure 1). These results agree with those obtained by Amores-Arrocha et al. (2018) [18] for the alcoholic fermentation of white grape musts, where a linear correlation was also observed between the bee pollen doses applied and the maximum viable yeast cells observed (R^2^ = 0.86). Results also agree with those obtained by Roldán et al. (2011) [22] for mead fermentation, where it was also concluded that bee pollen is a correct activator of the alcoholic fermentation process. The bee pollen activating effect on flor velum yeasts during biological aging may also be because it provides easily assimilable substances and growth factors such as amino acids, vitamins, and micronutrients [20]. Different authors indicate that bee pollen YAN is mainly due to its amino acids, representing 14–16% of YAN total content, especially proline, glutamic acid, aspartic acid, lysine, and leucine [20]. According to Roldán et al. (2011) [22], proline is the most important free amino acid in bee pollen composition and it is found in a 15.5 mg/g concentration. Dos Santos et al. (2000) [25] showed that during biological aging there is a reduction in the L-proline content since it is one of the main nitrogen sources consumed by flor velum yeasts. According to Berlanga et al. (2006) [10], glutamic acid and leucine can activate flor yeast development. All these amino acids may have a positive influence on flor velum development acting as a direct source for the synthesis of cellular proteins. Our results confirm that bee pollen addition implies a significant increase in YAN in wines at the beginning of the trial, and this fact may be one of the essential factors of flor velum yeast activation. However YAN consumption is not proportional to the maximum population reached during biological aging (Figure 3). It was expected that at high bee pollen doses, where a greater flor velum yeast growth was granted, YAN decrease should be greater. This fact could indicate that bee pollen could solubilize YAN throughout the process, especially in the initial growth phase, when the alcohol content is higher (15% approximately). Some authors have verified that YAN extraction in bee pollen increases with the alcoholic degree [18]. This effect was also observed during grape must alcoholic fermentation, but in this case, the maximum reduction reached with bee pollen addition was lower (20%) in respect to the control wine [18] and compared to the 60% achieved in our trials. In this way, this fact could indicate that bee pollen addition to wine could reduce the YAN requirements during biological aging since pollen supplies other growth-activating substances, such as fatty acids [26,27]. However, if is taken into account that pollen is a YAN reserve source, an increase in YAN content would be expected during the stationary phase for all the samples. This did not happen possibly because YAN consumption was still very high, due to an increase in flor velum populations and also because the YAN extraction effect is not favored as a result of a decrease in the alcoholic strength during this phase. Additionally, the YAN increase observed at the end of the biological aging could be favored by the yeast autolysis phenomenon in which an important amount of amino acids and other nitrogenized substances are released to the wine [28]. At the end of the biological aging, YAN content could be beneficial, for example, in case the flor velum has to be implanted again in a wine cask, in cases of cell death due to high temperatures, or to promote the flor velum development in very old Sherry wines, which have difficulties in keeping flor velum alive. 

Regarding to the increase observed in hydrophobicity levels (Figure 4), this fact may be because multiflora bee pollen can provide some fatty acids responsible for the yeast cell wall hydrophobicity [29]. Aguilera et al. (1997) [30] found that oleic or palmitic acid contribution to the flor velum allows a greater cellular buoyancy. In this sense, some authors have verified that bee pollen has a high content of oleic and palmitic acids that represent approximately the 40% of the fatty acids total concentration [21,31]. Therefore, bee pollen is an input if substrates that allow the synthesis of compounds increasing cell surface hydrophobicity and the ability to float in a filmogenic phase over the wine.

In respect to the flor velum yeast metabolism (Table 1), the slight increase in pH for the 20 g/L bee pollen dose could be due to cation exchange, mainly potassium [21]. Ethanol (alcoholic strength) is the main metabolite consumed by flor velum yeasts [32], and it was expected to decrease at a greater rate, especially at high bee pollen doses (10–20 g/L) where the yeast populations were higher. However, this did not come into existence, even though the wine alcoholic strength for 10 and 20 g/L doses were higher than in the control. It is necessary to bear in mind that in our trials the ethanol decrease was due mainly to the evaporation phenomena that depend on many factors. Perhaps, velum with larger populations can wield a protective effect against the ethanol evaporation. Regarding volatile acidity, the slight variation observed for the samples may be a result of the limited growth of lactic acid bacteria present naturally in industrial velums [33,34] and/or production of acetic acid from acetaldehyde as a yeast metabolic regulator [35].

In relation to the organic acids composition, the presence of citric acid in such a low concentration is because it is a citric acid cycle substrate that is subsequently metabolized into ketoglutaric acid [7]. The slight tartaric acid concentration decrease may be associated with the pollen dose due to the potassium contribution by the pollen to the wine, which could cause the precipitation of this acid in the form of potassium salts [36]. Regarding the malic acid content, which is a fundamental substrate of lactic acid bacteria, no significant differences were observed between the different samples, and therefore it is deduced that the wines have not undergone any malolactic fermentation processes [37].

Referring to the major volatile compounds concentration, acetaldehyde is recognized as one of the main products in the biological aging process [38] and it is produced by the oxidative metabolism of ethanol [15]. However, its formation is influenced by the wine conditions [35] and especially by the major yeast strain present in the velum [39]. It has been found that the yeast strain employed in this research work is less tolerant than other *S. cerevisiae* strains to acetaldehyde, so when its concentration is high, generally above 200 mg/L, flor velum yeasts metabolize it through pathways like the Krebs or glyoxilate cycle. Thus, the acetaldehyde values presented by wines supplemented with pollen at the end of the biological aging are normal considering the *Saccharomyces cerevisiae* strain used in this study [38]. This yeast strain can also form acetic acid in order to reduce the medium toxicity. This fact may justify that the acetaldehyde levels in this study present oscillations depending on whether the yeast strain is in a predominant consumption or production phase. Regarding ethyl acetate concentration, bee pollen addition to wines is an input of fatty acids and amino acids [19]. In this way, bee pollen could be able to favor the esterification process to form ethyl acetate [40] through a major activation of acetyl transferase and esterase activities [41]. In respect to the methanol content, Amores-Arrocha et al. (2018) [42] obtained similar results. The increase observed could be associated with the high pectin content of bee pollen [22]. Some authors have confirmed that pectins can be solubilized and metabolized by yeasts during alcoholic fermentation, outputting methanol through an enzymatic hydrolysis [21]. In this way, is probably that this enzymatic mechanism is also present in flor velum yeasts during biological aging. Major alcohol content depends on the temperature and amino acid concentration among other factors. The major alcohols present in samples may be due to the Ehrlich pathway catabolic process, where amino acids act as precursors of the major alcohol synthesis [43]. Concretely, the increase in isobutanol and isoamyl alcohol content is favored by the presence of valine and leucine in the medium [44]. According to Roldán et al. (2011) [22] leucine and valine represent approximately 2% of the total amino acids present in bee pollen, thus, bee pollen addition to wines for further biological aging could increase the content of major alcohols. Despite of that, the contribution of amino acids in high doses of pollen has not produced a higher content of major alcohols. This fact could be due to a metabolic regulation since the absence of oxidized cofactors like NAD^+^ would prevent the transformation of the intermediate aldehyde into major alcohols [40]. Glycerol is the second source of carbon consumed by flor velum yeasts during biological aging and nowadays it can be considered as the key compound for the flor velum metabolism analysis [35]. Glycerol consumption is not affected by the presence of bee pollen, except for the 20 g/L dose where its consumption is higher. This fact could indicate that only high doses of pollen can increase flor velum metabolism. Therefore, flor velum metabolism is not significantly affected by the presence of pollen or by its positive effect on yeast growth rate.

Regarding the results obtained from the sensory analysis (Figure 5a,b), bee pollen addition can improve the sensory attributes of biological aging in both the olfactory (dried fruit, yeast/bread, floral) and taste phase (salinity flavor and dryness sensation) when is applied in a concentration under 1 g/L. From this dose on, and specially for high doses (10–20 g/L), wines lose its characteristic sensory attributes, and acquire sensory deviations like an increase in color intensity and cereal/pollen aroma that may affect wine final quality. This color increase could be associated with the polyphenols granted by bee pollen [19] that could ease oxidation reaction development in wine in the presence of oxygen and, as a consequence, destabilize the final wine sensory quality.

## 4. Materials and Methods

### 4.1. Velum Yeast

Flor velum yeast was obtained from a biological aging system of a Sherry wine cellar and was identified as *Saccharomyces cerevisiae beticus,* a strain identified morphologically and codified as ‘‘B16” by Martínez (1995) [39]. The velum yeasts were disaggregated using a P-Selecta ultrasonic bath (Barcelona, Spain) and homogenized in a small volume of wine to be subsequently inoculated at a known concentration using a submerged protocol [33].

### 4.2. Fino-Sherry Wine

Fino-Sherry from Palomino Fino grapes (*Vitis vinifera*) was obtained from a 2 years old “criadera” from a “criadera y solera” aging system of the Andalusian winery “Cooperativa Unión de Viticultores Chiclaneros”, Chiclana de la Frontera (Cadiz, Spain). Wine was filtered using a Whatman 0.2 µm membrane filter (Darmstad, Germany) to eliminate all suspended particles and/or microorganisms and characterized physicochemically before the biological aging trials began (initial sample).

### 4.3. Bee Pollen

Commercial multiflora bee pollen (Valencia, Spain) was grounded in a Vowerk’s Thermomix TM31 mill (Wuppertal, Germany), sterilized under ultraviolet light for 2 h, and stored in the dark under dessicator conditions [18].

### 4.4. Effect on Flor Velum Yeast Growth

To study the influence of bee pollen addition on yeast development in biologically aged wines, commercial bee pollen was added to 10 mL of “Fino” sherry wine inside test tubes using six different doses: 0 (control), 0.1, and 0.25 g/L (low doses), 1 and 5 g/L (intermediate doses), and 10 and 20 g/L (high doses). Bee pollen was dissolved in wine using a P-Selecta ultrasonic bath (Barcelona, Spain). Then, yeast was inoculated at a 4.8 × 10^3^ ± 650 CFU/mL concentration following a submerged protocol [33]. Test tubes were stored in a temperature-controlled area at 20 ± 1 °C for 27 days using a P-Selecta lab refrigerator (Barcelona, Spain). Each bee pollen dose and control sample was conducted in triplicate (n = 3) to ensure statistical significance.

#### Analytical Measurements

Yeast population counts were performed using an optical Nikon microscope with 40× magnification (Tokyo, Japan) with the methylene blue staining method in a Merck Neubauer chamber (Madrid, Spain). Hydrophobicity was assessed using the toluene method [29]. Harvested flor velum yeasts cells were washed with water and suspended in a pH = 3.5 buffer, adjusting cell population as the optical density at λ = 660 nm reached 0.5. Three milliliters of this suspension was placed into another test tube and the equivalent volume of organic solvent was gently put upon the buffer. The tube was vigorously shaken using a Heidolph shaker (Schwabach, Germany). The optical density of the initial and residual buffer solution was measured. Hydrophobic degree (HD) of the yeast cell surface was calculated from the equation: HD (%) = 100 × (1−R/I); where R and I were the optical density of the initial and residual buffer solution respectively. Yeast assimilable nitrogen (YAN) was determined according to the formaldehyde method with modifications described by Aerny (1997) [45]. All the measurements were destructive analysis and were conducted in triplicate to ensure statistical significance.

### 4.5. Effect of Bee Pollen on Flor Velum Metabolism

To identify bee pollen addition influence on flor velum yeast metabolism during biological aging, oenological parameters, major volatile compounds, and organic acids were assessed in all samples. Flor velum at a concentration of 7.4 × 10^4^ ± 114 CFU/mL was inoculated in 200 mL of bee pollen supplemented wine inside Greiner Bio-One flasks (Kremsmünster, Germany) stored in the same conditions as the test tubes noted in Section 2.3. Under the same conditions, 200 mg/L of potassium sorbate (Sigma–Aldrich, Saint Louis, MO, United States) were added to another 200 mL flask of wine to control the ethanol amount evaporated during the biological aging process. All the experiments were conducted in triplicate. Once the experiment was finished, wines were filtered using 0.2 µm membrane filters (Darmstad, Germany) and bottled using an inert atmosphere (N_2_) to avoid changes until their characterization and sensory analysis.

#### Analytical Measurements

Total acidity, volatile acidity, and alcohol content were determined according to the official methods of wine analysis [46]. pH determinations were carried out using a Crisson digital pH-meter (Loveland, CO, United States). Glycerol was determined using a Biosystems enzymatic kit (Barcelona, Spain) with a Thermo–Fischer UV-Vis spectrophotometer (Whaltman, MA, United States). Major volatile compounds (acetaldehyde, ethyl acetate, methanol, 1-propanol, isobutanol, and isoamyl alcohol) were determined using the method proposed by Amores-Arrocha et al. (2018) [41]. A 5 µL direct injection in an Agilent Technologies HP 5890 gas chromatograph equipped with a FID detector (Santa Clara, CA, United States) on a Sigma–Aldrich Carbowax 20M column (L 50m, ID 0.25mm, PD 0.25 µm) (Saint Louis, MO, United States). Injector temperature was 175 °C and detector temperature was 225 °C. Hydrogen was used as a carrier gas with a 1 mL/min flow. Oven temperature was set at 35 °C for the first 5 min with a 5 °C/min ramp until 100 °C were reached. 4-metil-2-propanol was used as internal standard at 783 mg/L concentration, and Sigma–Aldrich pure standard compounds (Saint Louis, MO, United States) were used to determine retention times and calibration curves. Citric, tartaric, malic, succinic, and lactic acid were assessed through ionic chromatography in a Metrohm 930 compact IC Flex ionic chromatograph equipped with a conductimetric detector on a Metrosep Organic Acids column -250/7.8- (Herisau, Switzerland). Organic acid separation was performed using as eluent a H_2_SO_4_ 0.4 mM in a 12% acetone solution at an isocratic 0.4 mL/min flow.

### 4.6. Sensory Analysis

Sensory analysis was performed to find differences among the different bee pollen doses applied to wine. Three days after bottling, a 10-member expert panel in individual booths with controlled illumination performed sensory analysis. Fifty milliliters of wine were served to each taster in standard tasting glasses [47]. Each taster was given specific tasting notes to evaluate on a 10 points scale, visual (color intensity), olfactory (floral, fruity, dried fruits, etc.), and taste (saltiness, sweetness, dryness, etc.) attributes, as well as the wine general assessment. The attributes evaluated were selected according to Jackson (2002) [48].

### 4.7. Statistical Analysis

Means and standard deviations were calculated and significant differences were evaluated by two-way ANOVA and Bonferroni’s multiple range (BSD) test with a *p* < 0.05 (GraphPad Prism version 6.01 for Windows, GraphPad Software, San Diego, CA, United States).

## 5. Conclusions

Bee pollen can be an effective flor velum activator in biologically aged wines when added in concentrations equal or higher than 0.25 g/L. It can reduce flor velum latency times, increasing yeast population growth rate according to the bee pollen dose. This effect may be due to the bee pollen YAN contribution to wines. During the yeast growth phase, YAN consumption per colony forming unit is significantly reduced with the pollen dose applied, possibly due to a decrease in YAN requirements by yeasts or to the fact that pollen continues releasing nitrogenized compounds throughout the biological aging process. Furthermore, bee pollen can improve and activate flor velum yeast surface hydrophobicity and its buoyancy during this aging process.

Generally, except for the 20 g/L dose, bee pollen addition does not produce any significant effect on flor velum metabolism, physicochemical parameters, organic acids content, major volatile compounds, or glycerol. The descriptive sensorial analysis determined that low bee pollen doses (0.1–0.25 g/L) increase biological aging attributes (dried fruit, yeast/bread, floral, salinity flavor, and dryness sensation), obtaining the best evaluation by the tasting panel. In this way, can be concluded that bee pollen can be a viable and natural tool to boost flor velum yeast development during biological aging and to improve the sensorial quality of final wines.

## Figures and Tables

**Table 1:** Effect of bee pollen on the physicochemical parameters and the total organic acids and major volatile compounds content.

Parameter	Before Biological Aging	After Biological Aging							
	Initial Sample	Control		0.1 g/L	0.25 g/L	1 g/L	5 g/L	10 g/L	20 g/L
pH	3.110 ± 0.014 ^a^	2.870 ± 0.02 ^a^		2.950 ± 0.01 ^a^	2.970 ± 0.03 ^a^	3.000 ± 0.15 ^a^	3.000 ± 0.08 ^a^	3.060 ± 0.01 ^a^	3.160 ± 0.05 ^a^
% Alcohol *v*/*v*	15.015 ± 0.064 ^a^	12.555 ± 0.06 ^b^		13.010 ± 0.06 ^b^	13.010 ± 0.24 ^b^	12.760 ± 0.015 ^b^	12.790 ± 0.08 ^b^	12.875 ± 0.12 ^b^	12.975 ± 0.177 ^b^
Total acidity (g/L)	5.870 ± 0.070 ^a^	6.030 ± 0.040 ^a^		5.990 ± 0.060 ^a^	5.840 ± 0.060 ^a^	5.840 ± 0.060 ^a^	5.720 ± 0.080 ^a^	5.920 ± 0.010 ^a^	6.020 ± 0.050 ^a^
Volatile acidity (g/L)	0.270 ± 0.050 ^a^	0.300 ± 0.010 ^a,d^		0.220 ± 0.010 ^b^	0.200 ± 0.010 ^b^	0.160 ± 0.010 ^c^	0.270 ± 0.010 ^a^	0.320 ± 0.010 ^d^	0.370 ± 0.010 ^e^
Citric acid (mg/L)		0.015 ± 0.001 ^a^		0.015 ± 0.001 ^a^	0.016 ± 0.001 ^a^	0.016 ± 0.001 ^a^	0.019 ± 0.001 ^a,b^	0.024 ± 0.001 ^b,c^	0.029 ± 0.001 ^c^
Tartaric acid (g/L)	2.166 ± 0.001 ^a^	2.018 ± 0.016 ^a^		2.070 ± 0.002 ^a^	2.025 ± 0.002 ^a^	2.089 ± 0.016 ^a^	1.977 ± 0.005 ^a^	1.976 ± 0.007 ^a^	1.677 ± 0.017 ^a^
Malic acid (mg/L)	0.226 ± 0.001 ^a^	0.240 ± 0.001 ^a^		0.244 ± 0.001 ^a^	0.240 ± 0.001 ^a^	0.251 ± 0.003 ^a^	0.253 ± 0.001 ^a^	0.260 ± 0.002 ^a^	0.273 ± 0.003 ^a^
Succinic acid (mg/L)	0.450 ± 0.003 ^a^	0.285 ± 0.001 ^b^		0.272 ± 0.001 ^b^	0.276 ± 0.002 ^b^	0.263 ± 0.002 ^b^	0.208 ± 0.002 ^b^	0.199 ± 0.003 ^b^	0.194 ± 0.001 ^b^
Lactic acid (mg/L)	0.109 ± 0.005 ^a^	0.013 ± 0.007 ^b^		0.003 ± 0.001 ^c^	0.010 ± 0.001 ^b,c^	0.018 ± 0.001 ^b,d^	0.021 ± 0.001 ^d^	0.036 ± 0.001 ^e^	0.024 ± 0.002 ^d^
Acetaldehyde (mg/L)	77.648 ± 9.900 ^a^	135.344 ± 2.806 ^b^		184.024 ± 3.689 ^c^	164.583 ± 8.479 ^d^	207.665 ± 8.339 ^e^	182.735 ± 6.958 ^c^	155.533 ± 5.505 ^d^	92.824 ± 2.135 ^a^
Ethyl acetate (mg/L)	38.275 ± 2.167 ^a^	52.705 ± 4.276 ^b^		47.761 ± 1.450 ^b^	50.462 ± 4.731 ^b^	53.870 ± 6.109 ^b^	53.304 ± 7.496 ^b^	66.499 ± 4.032 ^c^	77.285 ± 6.590 ^d^
Methanol (mg/L)	35.039 ± 1.247 ^a^	33.286 ± 2.702 ^a^		43.978 ± 2.844 ^b^	46.786 ± 2.468 ^b^	53.726 ± 1.960 ^c^	58.153 ± 1.249 ^c^	57.755 ± 2.789 ^c^	58.286 ± 1.099 ^c^
1-Propanol (mg/L)	19.718 ± 1.287 ^a^	29.463 ± 2.484 ^b^		27.464 ± 4.640 ^b^	34.342 ± 2.499 ^c^	42.104 ± 2.475 ^d^	42.596 ± 3.241 ^d^	30.325 ± 2.221 ^b^	20.662 ± 1.767 ^a^
Isobutanol (mg/L)	32.222 ± 1.329 ^a,d^	34.755 ± 1.734 ^a,e^	43.989 ± 0.145 ^b^		39.421 ± 1.496 ^b,e^	58.307 ± 1.334 ^c^	61.690 ± 1.775 ^c^	29.241 ± 1.429 ^d^	29.247 ± 2.484 ^b^
Isoamyl alcohol (mg/L)	207.100 ± 7.838 ^a^	215.534 ± 5.815 ^a^	246.114 ± 1.160 ^a,b^		256.654 ± 6.791 ^a,b^	263.513 ± 4.338 ^a,b^	290.323 ± 0.845 ^b^	235.421 ± 3.881 ^a,b^	201.665 ± 5.627 ^a^
Glycerol (mg/L)	1624.240 ± 0.03 ^a^	97.830 ± 3.34 ^b^		99.910 ± 9.34 ^b^	97.850 ± 6.67 ^b^	100.410 ± 6.00 ^b^	92.460 ± 7.34 ^b^	83.300 ± 6.27 ^b^	64.000 ± 6.00 ^c^

Different uppercase letters mean statistically significant differences between samples at *p* < 0.05 obtained by two-way ANOVA and Bonferroni’s multiple range (BSD) test. Results are the means ± SD of three repetitions.
